# *Aggregatibacter actinomycetemcomitans* Biofilm Reduces Gingival Epithelial Cell Keratin Expression in an Organotypic Gingival Tissue Culture Model

**DOI:** 10.3390/pathogens8040278

**Published:** 2019-12-01

**Authors:** Arzu Beklen, Annamari Torittu, Riikka Ihalin, Marja Pöllänen

**Affiliations:** 1 Department of Biochemistry, University of Turku, 20014 Turku, Finland; arzu.beklen@gmail.com (A.B.); atorittu@abo.fi (A.T.); riikka.ihalin@utu.fi (R.I.); 2 Institute of Dentistry, University of Turku, 20014 Turku, Finland

**Keywords:** *Aggregatibacter actinomycetemcomitans*, keratins, organotypic gingival mucosa

## Abstract

Epithelial cells express keratins, which are essential for the structural integrity and mechanical strength of the cells. In the junctional epithelium (JE) of the tooth, keratins such as K16, K18, and K19, are expressed, which is typical for non-differentiated and rapidly dividing cells. The expression of K17, K4, and K13 keratins can be induced by injury, bacterial irritation, smoking, and inflammation. In addition, these keratins can be found in the sulcular epithelium and in the JE. Our aim was to estimate the changes in K4, K13, K17, and K19 expression in gingival epithelial cells exposed to *Aggregatibacter actinomycetemcomitans.* An organotypic gingival mucosa and biofilm co-culture was used as a model system. The effect of the biofilm after 24 h was assessed using immunohistochemistry. The structure of the epithelium was also studied with transmission electron microscopy (TEM). The expression of K17 and K19, as well as total keratin expression, decreased in the suprabasal layers of epithelium, which were in close contact with the *A. actinomycetemcomitans* biofilm. The effect on keratin expression was biofilm specific. The expression of K4 and K13 was low in all of the tested conditions. When stimulated with the *A. actinomycetemcomitans* biofilm, the epithelial contact site displayed a thick necrotic layer on the top of the epithelium. The *A. actinomycetemcomitans* biofilm released vesicles, which were found in close contact with the epithelium. After *A. actinomycetemcomitans* irritation, gingival epithelial cells may lose their resistance and become more vulnerable to bacterial infection.

## 1. Introduction

Periodontal disease is an inflammatory disease caused by growing biofilm, which gradually develops to a bacterial community rich in inflammophilic Gram-negative species [1]. The formation of such biofilm and the subsequent inflammation affects the supporting tissues of the tooth, including the epithelium and the connective tissue. Cytoskeletal intermediate filaments are essential for the structural integrity and function of the epithelial cells. Intermediate filaments constitute the acidic (Type I) and basic (Type II) keratins. The molecular weight of Type I keratin ranges from 40 to 57 kDa and encompasses keratins K9–K20. The molecular weight of Type II keratin ranges from 50 to 70 kDa and encompasses keratins K1–K8.

Keratins play an important functional role in the integrity and mechanical stability of both individual epithelial cells and cell–cell contacts in tissues. Thus, they contribute not only to the stability of the epithelium itself but also to the basement membrane attachment to the connective tissue underlying the epithelium. In the epithelia of internal organs, which are under little mechanical stress, there are only a few loosely-distributed keratin filaments in the cytoplasm. Conversely, keratins are abundant and are densely bound in the lining of outer surfaces. The composition of keratins varies depending on the epithelial cell type and the differentiation status of the epithelial cells. The keratin composition may also be affected by external stimuli, inflammation, or other types of disease development (e.g., cancer). The oral epithelium is a keratinizing form of epithelium and provides an effective physical barrier to microbial invasion. In the oral epithelium, basal dividing cells express simple epithelial cell keratins (K5, K14, and K19), whereas suprabasal cells express keratins typical of differentiated cells (K1, K10, K6, and K16). The keratin profile of the junctional epithelium (JE) differs from the oral epithelium, as only keratins typical for non-differentiated and rapidly dividing cells are expressed both basally (K5, K14, and K19) and suprabasally (K19). In addition, keratins such as K4, K13, and K17 have been found in the sulcular area of the dentogingival junction and may be induced by acute injury, bacterial irritation, smoking, and inflammation [2,3]. 

In contrast, in patients with severe and rapidly progressive periodontitis (previous term aggressive periodontitis), K17 gene expression was found to be repressed in disease site gingival samples compared to healthy site samples [4]. An open issue of significant interest is whether the expression of those keratins is altered by irritation from biofilm bacteria, such as *Aggregatibacter actinomycetemcomitans*, in periodontal diseases. The Gram-negative bacterium *A. actinomycetemcomitans* is an aggressive pathogen that is frequently associated with subgingival biofilms and has been strongly implicated in the development of rapidly progressive periodontal disease involving the invasion of *A. actinomycetemcomitans* into epithelial layers. Although *A. actinomycetemcomitans* represents only one species in multispecies periodontal biofilm, it most likely is able to suppress the host defense with its virulence factors. To gain insight into this question, we examined keratin K4, K13, K17, and K19 expression and distribution in an organotypic gingival tissue culture model co-cultured with a periodontopathogenic *A. actinomycetemcomitans* biofilm. Keratin K19 was chosen since it is typical and most dominant keratin in the JE [5,6] and we wanted to investigate how well the tissue culture model mimics the JE. We found that the expression of K17 and K19, as well as total keratin expression, decreased in the suprabasal layers of epithelium, which were in close contact with the *A. actinomycetemcomitans* biofilm. The decreased keratin expression may lead to decreased resistance of gingival epithelial cells to bacterial infection.

## 2. Results

### 2.1. Control Cultures Showed Strong Expression of K17 and K19 and Only Weak or No Expression of K4 and K13

**Figure 1:**
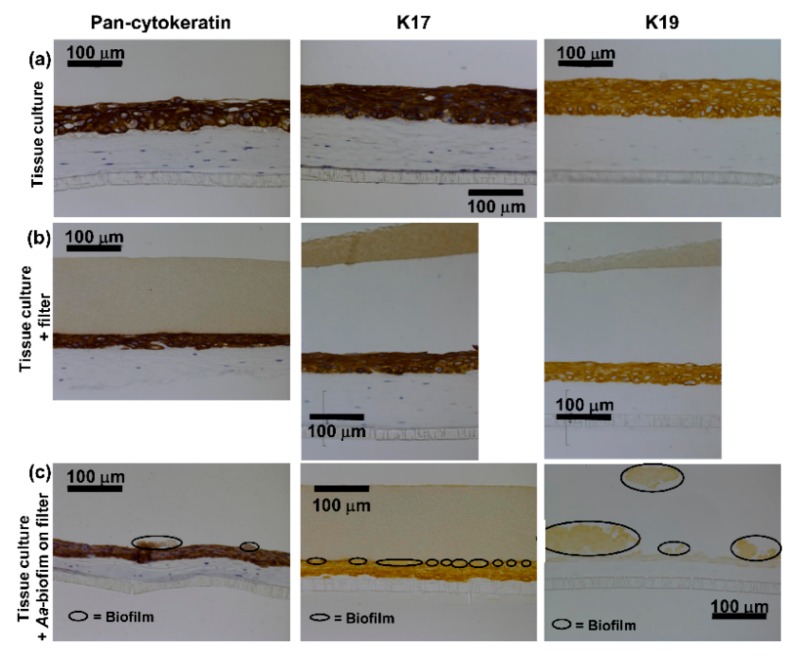
*A. actinomycetemcomitans* biofilm decreased the total keratin as well as K17 and K19 expression in the suprabasal layers of the epithelium. (**a**,**b**) Immunohistochemical staining (peroxidase-3,3′-diaminobenzidine (DAB)) with anti-pan-cytokeratin/anti-cytokeratin 17/anti-cytokeratin 19 shows strong staining in the control cultures. (**c**) Pan-cytokeratin/K17/K19 expression is decreased in the co-cultures with the *A. actinomycetemcomitans* biofilm.

**Figure 2:**
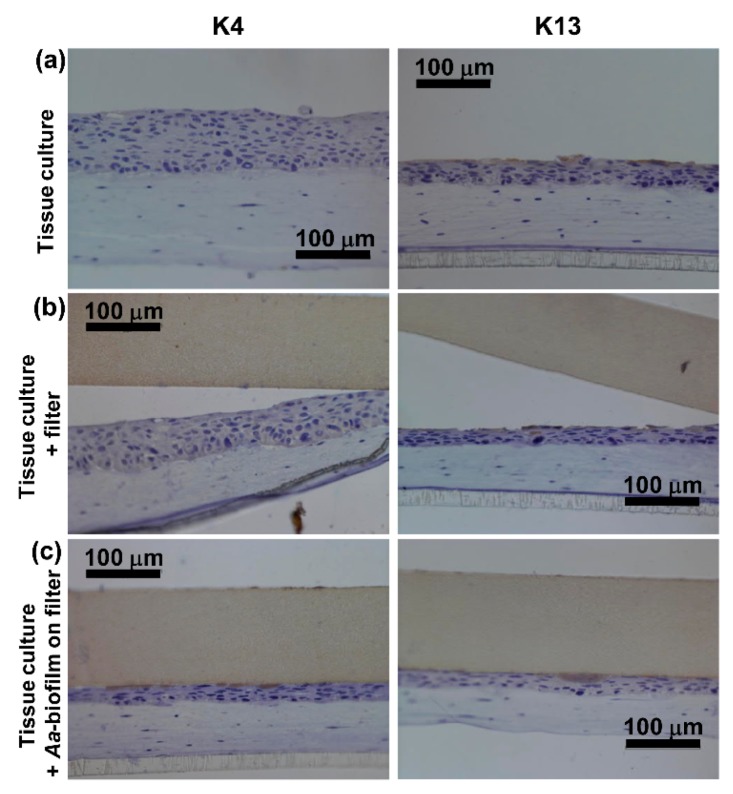
The expression of K4 and K13 was low in all of the tested conditions. (**a**,**b**) Immunohistochemical staining (DAB) with anti-cytokeratin 4 in the control cultures or (**c**) in the co-cultures with the *A. actinomycetemcomitans* biofilm shows no expression of K4. (**a**,**b**) Immunohistochemical staining (DAB) with anti-cytokeratin 13 shows only a few stained cells in the control cultures. (**c**) In the co-cultures with *A. actinomycetemcomitans* biofilm, no expression of K13 is observed.

Both control cultures grown without anything on the top of the epithelium or with an empty sterile filter disc showed similar immunohistochemical staining. Pancytokeratin staining was strong and was evenly distributed throughout the epithelium (Figure 1a,b). Similarly, the specific cytokeratins K17 and K19 were found to be expressed from the basal layer throughout the epithelium to the surface (Figure 1a,b). Keratin 4 was not found in the control cultures (Figure 2a,b), and K13 expression was only observed occasionally in single cells (Figure 2a,b).

### 2.2. A. actinomycetemcomitans Biofilm Decreased the Expression of Keratin 17 and 19, Which Was in Accordance with the Decreased Expression of Total Keratin

When exposed to pre-grown *A. actinomycetemcomitans* biofilm for 24 h, the human gingival keratinocytes showed no or very weak expression of K17 and K19 throughout the epithelium and especially in areas in close contact to the biofilm (Figure 1c). Some expression of K17 was evident further away from the biofilm-epithelium contact site (Figure 1c) as well as in the basal layer adjacent to the connective tissue. K19 expression was almost totally absent (Figure 1c). No expression of K4 or K13 was observed in the co-cultures with the biofilm (Figure 2c). The decrease in the expression levels of total keratins (pan-cytokeratin staining, Figure 1c) in the co-cultures with the *A. actinomycetemcomitans* biofilm was in accordance with the decrease in specific keratin expression.

### 2.3. A. actinomycetemcomitans Biofilm Caused Necrosis of the Epithelial Surface

**Figure 3:**
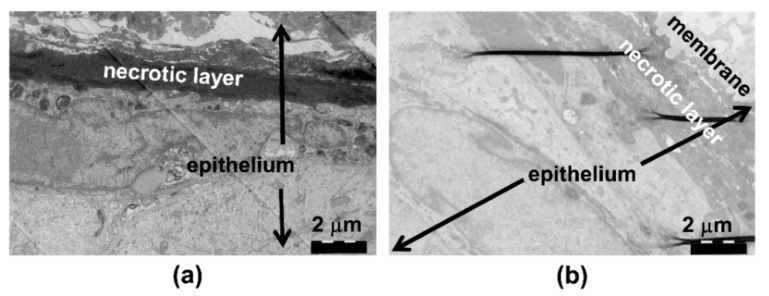
Normal structure of the epithelial cells and the epithelial cell nuclei. (**a**) Transmission electron microscopy (TEM) of the control cultures grown with nothing on the surface and (**b**) with the sterile membrane on the epithelium shows the normal structure of the cells and nuclei. On the top of the epithelium, a thin area of exfoliating and necrotic cells is observed (dark area).

**Figure 4:**
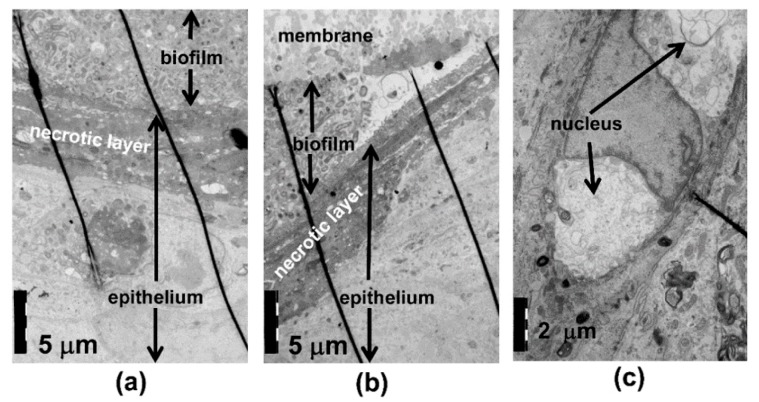
*A. actinomycetemcomitans* biofilm caused necrosis of the epithelial cells. (**a**,**b**) TEM of the co-cultures with *A. actinomycetemcomitans* shows the thickening of the necrotic layer in areas in close contact to the biofilm and (**a**,**c**) disruption of the nuclei beneath the biofilm.

**Figure 5:**
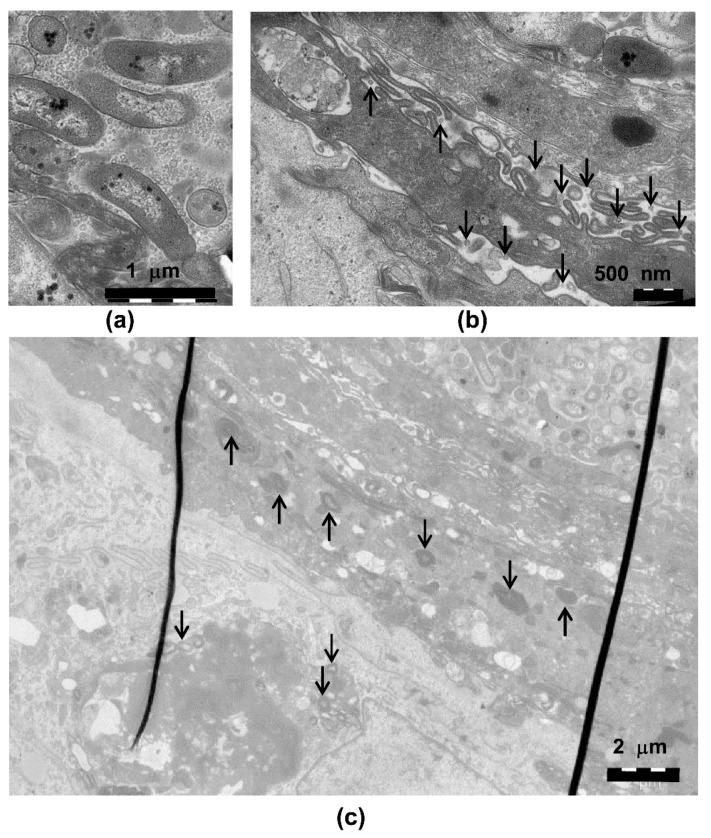
Vesicle-like structures are released by *A. actinomycetemcomitans* biofilm. (**a**) *A. actinomycetemcomitans* cells in the biofilm release vesicles, and (**b**) similar structures can be observed inside the epithelium. (**c**) (arrows) A few particles resembling single *A. actinomycetemcomitans* cells are observed inside the necrotic epithelium and inside the epithelial cells beneath the necrotic layer.

Using TEM analysis, the control culture surface showed a thin layer of exfoliating, necrotic cells (Figure 3a,b). The cell structures and cell–cell contacts appeared normal. When exposed to the *A. actinomycetemcomitans* biofilm, the necrotic area of the epithelial surface was thick (Figure 4a,b), and the thickness of the necrotic area increased in areas in close contact to the biofilm and with an increasing amount of biofilm (Figure 4b). Necrosis of the epithelium in contact with the biofilm was consistently seen in EM, as visualized in Figure 4b, where right beneath the increasing mass of *A actinonmycetemcomitans* (on the left side of the picture) the necrotic epithelial layer increases clearly in thickness. This is a descriptive result, but was consistenly seen in EM. In the co-cultures, nuclear breakdown was observed in areas in close contact to the biofilm (Figure 4a,c). When in close contact with the epithelium, the biofilm bacteria released vesicles, and similar structures could be observed intraepithelially (Figure 5a,b). The necrotic area appeared to act as a barrier to the biofilm bacteria; however, a few structures which resembled bacteria were observed inside the epithelium after the 24 h co-culture (Figure 5c).

## 3. Discussion

Our major findings were that the *A. actinomycetemcomitans* biofilm caused decreased expression of cytokeratins, which is typical for the dentogingival junction and necrosis of the epithelial surface layers in close contact to the biofilm. Our tissue culture model appeared to mimic JE well, as K19, the typical keratin for JE, was highly expressed in the control cultures [6]. Our finding of decreased K19 expression adjacent to the *A. actinomycetemcomitans* biofilm is in disagreement with earlier reports showing that the inflammation in the periodontal pocket increases K19 expression [3,7]. However, our result may reflect the specific nature of the *A actinomycetemcomitans* biofilm–host tissue interaction. Previous work has shown that *A. actinomycetemcomitans* can invade buccal epithelial cells [8] and gingival tissue [9]. By decreasing the expression of one major structural protein, K19, in JE cells, *A. actinomycetemcomitans* could disturb the epithelial integrity, ease the invasion of the bacteria into deeper tissues, and lead to subsequent periodontal connective tissue destruction. In fact, structures resembling single *A. actinomycetemcomitans* cells were observed in the TEM analysis of epitheliums of co-cultures. Tissue destruction may even be further accelerated by decreased epithelial cell proliferation, which we showed in a previous study of tissue cultures exposed to an *A. actinomycetemcomitans* biofilm [10].

K17 was also strongly expressed in our control cultures. In vivo, K17 has been found in the sulcular epithelium of the dentogingival junction [2]. In addition, JE seems to express the genes encoding K17 [11]. Furthermore, it has been previously suggested that short chain fatty acids produced by some Gram-negative periodontal pathogens increase the expression of K17 [2] and that the inflammatory mediators would play a role in the regulation of the K17 expression [12]. Our finding that an *A. actinomycetemcomitans* biofilm decreased the expression of K17 seems to conflict with the earlier studies showing increased K17 expression after treatment with short chain fatty acids [2]. However, *A. actinomycetemcomitans*, although resistant to the antimicrobial activity of short chain fatty acids [13], produces only long chain fatty acids [14], which may at least partly explain these contradictory findings. Furthermore, K17 gene expression has been found depressed in clinical specimens from patients with severe and rapidly progressive periodontitis, which is in agreement with our results [4].

The model showed no evidence of K4 or K13 expression, which have been shown to be expressed in healthy oral sulcular epithelium [15]. Although K4 is typically absent in JE, its expression can be observed in JE of smoking periodontitis patients [15]. In our model, the *A. actinomycetemcomitans* biofilm did not increase or decrease K4 or K13 expression, of which the latter has been shown increasingly expressed in inflamed tissues [3]. However, the increase in K13 expression in inflammation may require the presence of host immune cells, such as macrophages, which were not included to our tissue co-culture model.

A closer investigation of the epithelial surface structure by TEM revealed that *A. actinomycetemcomitans* biofilm exposure caused necrosis of the epithelial surface in the co-culture models. The thick necrotic layer most likely inhibited the invasion of *A. actinomycetemcomitans* cells into the deeper layers of epithelium, as we could only detect a few structures resembling *A. actinomycetemcomitans* cells in the surface layers of the epithelium. However, the *A. actinomycetemcomitans* biofilm secreted high amounts of vesicles, which could have greater invasive potential than whole bacterial cells. For instance, *A. actinomycetemcomitans* vesicles have been shown to invade human HeLa and gingival fibroblast cells and release cytolethal distending toxin to the host cell nucleus [16,17]. *A. actinomycetemcomitans* vesicle-like structures could be observed in the epithelium. However, these vesicles may also originate from the host epithelial cells, and thus their origin needs to be confirmed in further studies.

In conclusion, *A. actinomycetemcomitans* biofilm decreases the expression of K17 and K19 and the total expression levels of keratins in an organotypic gingival mucosa model. However, the epithelium appears to utilize additional measures to withstand attack by the *A. actinomycetemcomitans* biofilm, which is suggested by the thicker necrotic layer between the epithelial cells and biofilm. Whether host immune cells and macrophages, in particular, change the keratin expression pattern during biofilm attack requires further investigation in a more complex environment.

## 4. Materials and Methods 

### 4.1. A. actinomycetemcomitans Biofilm Culture

The *A. actinomycetemcomitans* biofilm cultures were generated as described previously [10]. Briefly, *A. actinomycetemcomitans* strain D7S was first cultured from trypticase soy agar blood plates. From the plates, an even bacterial suspension was made [18], and 5 × 10^7^ cells/well were added to a 48-well plate containing porous filter discs. The biofilms were first grown in a rich trypticase soy broth medium for 24 h on a filter disc, and after which they were washed with a 0.85% NaCl solution. The cultivation was continued for an additional 24 h in glutamine supplemented RPMI-1640 medium. The 48-well plate also contained wells with sterile filter discs in the appropriate media for use as controls.

### 4.2. Gingival Mucosa Co-Culture Models

The *A. actinomycetemcomitans* biofilm/gingival mucosa co-culture models were constructed as described previously [10]. Briefly, human gingival fibroblasts [19] were grown in a collagen suspension with a cell culture insert (ThinCert™, Greiner Bio-One GmbH, Germany) for one day before 4 × 10^5^ spontaneously immortalized human gingival keratinocyte cells [20] were seeded on top of the fibroblast-collagen matrix. The epithelial cells were cultured for one day submerged in the growth medium before the tissue model was lifted to the air–liquid interface. The model was air exposed for five days before a separately cultured *A. actinomycetemcomitans* D7S [21] biofilm (see above) was added on top of the tissue culture. The co-cultures were incubated without antibiotics in the cell culture medium. The gingival mucosa was co-cultured with the biofilm/control disc/no added components for 24 h, and the co-cultures were the fixed with a 10% formalin solution overnight. After the fixation step, the samples were embedded in paraffin and were sectioned.

### 4.3. Immunohistochemical Staining of Keratins K4, K13, K17, and K19

Before staining, the specimens were deparaffinized and heat-mediated antigen retrieval in 10 mM citrate buffer (pH 6.0) with microwaving was performed, which was followed by proteinase K treatment [22]. The staining was performed with a Dako TechMate™ 500 Plus Autostainer (Dako, Glostrup; Denmark) using the primary antibodies listed in Table 1 and the Dako REAL™ Detection System, Peroxidase/DAB+, Rabbit/Mouse (Code K5001; Dako) according to manufacturer’s instructions.

### 4.4. Transmission Electron Microscopic (TEM) Studies of the Biofilm-Epithelium Contact Site

The *A. actinomycetemcomitans* biofilm gingival mucosa co-culture models were assembled as described above. The samples were prefixed for TEM with a freshly prepared 5% glutaraldehyde solution (5% glutaraldehyde, 0.16 M s-collidin-HCl buffer, pH 7.4) for at least 3 h at room temperature. After prefixation, the samples were washed with s-collidin-HCl buffer three times for 3 min each. Then, the samples were postfixed (1% OsO4, 1.5% K-ferrocyanide) for 2 h [23], washed with s-collidin buffer three times for 5 min each, and were dehydrated with ethanol (70% ethanol, 1 min at 4 °C; 96% ethanol, 1 min, at 4 °C; 100% ethanol, 30 min, at 4 °C; 100% ethanol, three times, 30 min each, at 20 °C). The dehydrated samples were embedded in epoxy using the following series: propylene oxide, two times, 15 min each; propylene oxide + epoxy resin + DMP (10:10:0.15), 2 h; epoxy resin + DMP (10:0.15), 12 h; epoxy resin + DMP (10:0.15), at 60 °C, 36 h. The sectioning of the samples was accomplished with an ultramicrotome to a thickness of approximately 70 nm. After sectioning, the samples were stained with uranyl acetate (1% uranyl acetate in pure water for 30 min) and were rinsed three times with pure water for 30 s. Finally, the samples were stained with lead citrate (0.3% lead citrate in pure water for 3 min) and were rinsed with water as in the previous step. The samples were examined with a JEM-1400 Plus Transmission Electron Microscope (JEOL USA, Inc., Peabody, MA, USA). 

## Figures and Tables

**Table 1:** Primary anti-keratin antibodies used in the study.

Keratin	Dilution	Host	Type	Code	Manufacturer
Keratin 4	1/10	mouse	monoclonal	MON3015-1	Sanbio, Holland
Keratin 13	1/50	mouse	monoclonal	MUB0340S	Nordic MUbio, Holland
Keratin 17	1/20	mouse	monoclonal	M704601-2	Dako, Denmark
Keratin 19	1/100	mouse	monoclonal	M088801-2	Dako, Denmark
Pan-cytokeratin 5,6,8,17,(19)	1/50	mouse	monoclonal	M0821	Dako, Denmark
